# Efficient and Stable Large-Area Perovskite Solar Cells with Inorganic Perovskite/Carbon Quantum Dot-Graded Heterojunction

**DOI:** 10.34133/2021/9845067

**Published:** 2021-07-12

**Authors:** Qiang Sun, Cai Shen, Deyu Wang, Tao Zhang, Huaxia Ban, Yan Shen, Zhipan Zhang, Xiao-Li Zhang, Guanjun Yang, Mingkui Wang

**Affiliations:** ^1^Wuhan National Laboratory for Optoelectronics, Huazhong University of Science and Technology, Wuhan, Hubei 430074, China; ^2^Ningbo Institute of Materials Technology & Engineering, Chinese Academy of Sciences, 1219 Zhongguan Road, Ningbo 315201, China; ^3^School of Chemistry, Beijing Institute of Technology, Beijing 102488, China; ^4^State Centre for International Cooperation on Designer Low-Carbon & Environmental Materials, School of Materials Science and Engineering, Zhengzhou University, 450001, China; ^5^State Key Laboratory for Mechanical Behavior of Materials, Xi'an Jiaotong University, Xi'an 710049, China

## Abstract

This work reports on a compositionally graded heterojunction for photovoltaic application by cooperating fluorine-doped carbon quantum dots (FCQDs in short) into the CsPbI_2.5_Br_0.5_ inorganic perovskite layer. Using this CsPbI_2.5_Br_0.5_/FCQDs graded heterojunction in conjunction with low-temperature-processed carbon electrode, a power conversion efficiency of 13.53% for 1 cm^2^ all-inorganic perovskite solar cell can be achieved at AM 1.5G solar irradiation. To the best of our knowledge, this is one of the highest efficiency reported for carbon electrode based all-inorganic perovskite solar cells so far, and the first report of 1 cm^2^ carbon counter electrode based inorganic perovskite solar cell with PCE exceeding 13%. Moreover, the inorganic perovskite/carbon quantum dot graded heterojunction photovoltaics maintained over 90% of their initial efficiency after thermal aging at 85° for 1056 hours. This conception of constructing inorganic perovskite/FCQDs graded heterojunction offers a feasible pathway to develop efficient and stable photovoltaics for scale-up and practical applications.

## 1. Introduction

Lead halide perovskites with the ABX_3_ formula (where X is halogen anion, A is monovalent cation (methylammonium (MA^+^), formamidinium (FA^+^) or Cs^+^), and B is Pb^2+^) have emerged as disruptive photovoltaic materials for solar energy conversion owning to a rapid increase in power conversion efficiency (PCE) [1–5]. However, the instability against thermal stress and humidity originated from the hygroscopic and volatile nature of organic cations (including MA^+^ and FA^+^) in these organic-inorganic hybrid perovskites as well as organic materials for collecting currents in a complete device has been the primary issue to hinder their commercialization [6–8]. Therefore, a solar cell free of organic components, which is constructed solely with inorganic perovskite compounds and inorganic functional materials including charge transport materials and contact electrode materials, offers great possibilities of enhancing solar cells performance with high stability.

In this regard, all-inorganic perovskite solar cells (PSCs) with carbon counter electrodes are thus developed in view of the structural stability and cost-effectiveness, and have become research interests arising from great prospect toward commercialization [9–14]. For example, an all-inorganic PSC with a structure of FTO/TiO_2_/CsPbIBr_2_/carbon has been reported by Zhu et al., which achieved a PCE over 9% and an open-circuit voltage (*V*_OC_) of 1.245 V [10]. Previously, we reported 10.8% efficient stable full-printable PSCs using an inorganic metal oxide framework of FTO/TiO_2_/Al_2_O_3_/NiO/carbon and inorganic perovskite CsPb_0.98_Mg_0.02_I_2_Br [15]. The inorganic perovskites featuring with excellent compositional and thermal stability offer promising potentials to process solar cells with high stability [16–18]. The utilization of moisture resistant carbon counter electrode offers additional advantages to inorganic PSCs, including good stability in ambient conditions and low-cost manufacture [19]. Moreover, the carbon electrode based PSCs can be fabricated with printing technology such as gravure printing and flexographic printing, which is beneficial for the scalable production of large modules [20]. Unfortunately, relatively lower PCE (typically 7%-11%) has been often observed for the inorganic PSCs using carbon counter electrode comparing to their counterparts that using 2,2′,7,7′-tetrakis(*N*,*N*-di-4-methoxyphenylamino)-9,9′-spiro-bifluorene (spiro-OMeTAD) as the hole-transport layer (HTL) and Au as the counter electrode [19, 21].

The interface between perovskite layer and carbon electrode is crucial to the photovoltaic performance of carbon electrode based PSCs. A poor interfacial contact as well as mismatched energy level alignment between perovskite layer and carbon electrode significantly induces undesirable charge recombination [22–25]. This also explains the widely observed large *V*_OC_ loss (defined as *E*_*g*_/*q* − *V*_OC_, where *E*_*g*_ is the bandgap of the absorption material, *q* is elementary charge), as well as low fill factor (FF) for carbon electrode based PSCs [9–14]. Considering the big resistance of carbon counter electrode (~20-40 *Ω*/□) with large-sized particles of high porosity, indeed, minimization of the interfacial recombination kinetics gives a good shot of enhancing *V*_OC_ along with FF. Therefore, in aim to enhance the photovoltaic performance of carbon electrode based all-inorganic PSCs, it is of fundamental importance to overcome the limits through the formation of intimate contact at the perovskite/carbon electrode interface and tailoring the interfacial energy-band alignment.

Graded-heterojunction (GHJ) engineering can be an effective method to facilitate the separation of photo-generated charge carriers and their transport in photovoltaic devices [26–30]. For example, an organic-inorganic hybrid perovskite-fullerene graded-heterojunction structure has been proposed by Wu and coworkers for the inverted-structured planar PSCs, which enabled the fabrication of centimeter-scale PSC device with a certified PCE exceeding 18% [26]. The carbon electrode-based PSCs with MAPbI_3_-PTAA gradient heterojunction also achieved a PCE of 13.0% in active area of 1 cm^2^ [29]. The graded-heterojunction architecture combines the advantage of short charge travel distance with the merit of introducing band slope at the heterojunction interface for transport of photo-generated charge carriers. Consequently, charge recombination loss can be minimized and thus augments the open-circuit voltage.

This work for the first time reports on compositionally graded heterojunction for photovoltaic application by injecting fluorine-doped carbon quantum dots (FCQDs in short) into inorganic CsPbI_2.5_Br_0.5_ perovskite *via* the antisolvent method. Because of the excellent optoelectronic properties together with high chemical stability, carbon quantum dots have shown significant potential as charge transport material or surface passivating agent in photovoltaic devices [31–34]. In this case, due to its small size and high polarization property, FCQDs carried by antisolvent (chlorobenzene) can be deposited into the CsPbI_2.5_Br_0.5_ perovskite layer before perovskite crystallization, forming a perovskite/FCQDs graded heterojunction with increasing FCQDs percentage along the vertical direction. The results indicate that the FCQDs interact strongly with CsPbI_2.5_Br_0.5_ perovskite and efficiently passivate the uncoordinated Pb ions defects. Moreover, the CsPbI_2.5_Br_0.5_/FCQDs graded heterojunction selectively promotes photo-generated charge separation and extraction, resulting in improved photovoltaic performance. The CsPbI_2.5_Br_0.5_/FCQD-graded heterojunction in conjunction with low-temperature-processed carbon electrode achieved an impressive power conversion efficiency of 13.53% in a 1 cm^2^ device at AM 1.5G solar irradiation. More importantly, the solar cells based on CsPbI_2.5_Br_0.5_/FCQDs GHJ in combination with low-temperature processed carbon electrode exhibited excellent long-term stability. The unencapsulated devices maintained over 90% of their initial efficiency under thermal aging at 85°С for 1056 h.

## 2. Results

**Figure 1:**
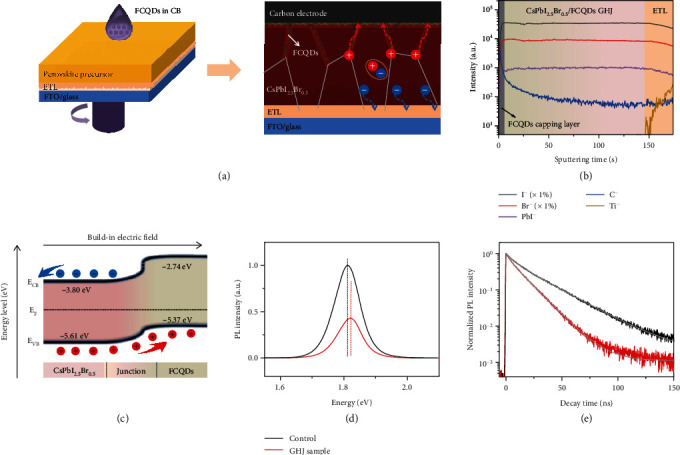
Material characterization of the graded heterojunction. (a) Schematic diagram for preparation of CsPb_2.5_Br_0.5_/FCQDs graded heterojunction devices. (b) TOF-SIMS depth profiles of CsPb_2.5_Br_0.5_/FCQDs graded heterojunction thin film. (c) Schematic diagram of band bending at the CsPbI_2.5_Br_0.5_ perovskite/FCQDs interface. Steady-state photoluminescence spectra (d) and time-resolved photoluminescence decay (e) curves of the CsPb_2.5_Br_0.5_ perovskite thin film and perovskite/FCQDs GHJ thin film on glass substrates.

The CsPbI_2.5_Br_0.5_/FCQDs graded heterojunction was fabricated by injecting FCQDs into inorganic CsPbI_2.5_Br_0.5_ perovskite layer *via* antisolvent method during perovskite precursor spin-coating process as illustrated in Figure 1(a). In this case, partial FCQDs in a size of about 3-7 nm (characterized with transmission electron microscopy, Figure S1) carried by antisolvent chlorobenzene (CB) can be deposited into the upper layer of CsPbI_2.5_Br_0.5_ perovskite film before crystallization, while most of FCQDs maintain on perovskite layer surface, forming a CsPbI_2.5_Br_0.5_/FCQDs graded heterojunction structure with increasing FCQDs percentage along the vertical direction. This GHJ conception was verified by time-of-flight secondary-ion mass spectrometry (TOF-SIMS) characterization as shown in Figure 1(b) [15, 35]. When sputtering through the CsPbI_2.5_Br_0.5_/FCQDs GHJ thin film by using Bi^3+^ ion beam, the SIMS characterization detects the signals of Pb, I, Br, and C in negative mass detection mode, in which C was used as an indicator of FCQDs. Obviously, the C depth profile (blue line) shows a steep decline at the perovskite/FCQDs GHJ film surface and negligible counting amount in the bulk. The decrease of carbon content along the vertical direction verifies a graded distribution of FCQDs in the CsPbI_2.5_Br_0.5_ perovskite absorption layer.

The interfacial band alignment around CsPbI_2.5_Br_0.5_ and FCQDs was estimated by ultraviolet photoelectron spectroscopy (UPS) and UV-visible absorption spectroscopy characterization (Figure S2a-S2c). The Fermi energy level (*E*_*F*_) of CsPbI_2.5_Br_0.5_ and FCQDs was calculated to be −4.36 and −4.74 eV, respectively (Figure S2d). The corresponding valence band maximum (*E*_VB_) of CsPbI_2.5_Br_0.5_ and FCQDs was further evaluated to be −5.61 eV and −5.37 eV, respectively. By taking into account of the valance band maximum, the conduction band minimum, and the Fermi level of both CsPbI_2.5_Br_0.5_ and FCQDs, upon contact, electron is depleted from CsPbI_2.5_Br_0.5_ to FCQDs, causing an upward band bending at the CsPbI_2.5_Br_0.5_/FCQDs interface. Base on this understanding, we built up an energy-level diagram for the perovskite/FCQDs GHJ in Figure 1(c), which schematically illustrates the upper shift valance band of CsPbI_2.5_Br_0.5_ at the heterojunction interface. In addition to concentration gradient, this kind of band bending supplies additional driving force for photogenerated hole extraction and restrains electron flowing back.

Figure S3 shows scanning electron microscopy (SEM) image of surface morphology of the as-prepared perovskite/FCQDs GHJ thin film. The corresponding films are very dense and smooth with few aggregates and pinholes. The X-ray photoelectron spectroscopy (XPS) characterization in Figure S4 presents the chemical states of the as-fabricated films. The binding energies of Pb(4f_7/2_) and Pb(4f_5/2_) at 138.2 eV and 143.05 eV for CsPbI_2.5_Br_0.5_ sample positively shift to higher values by 0.015 eV for CsPbI_2.5_Br_0.5_/FCQDs GHJ sample. This is usually caused with the existence of additional negative charges surrounding Pb ions [36–38]. In this work, we attributed this to the presence of fluorine in FCQDs, which provides extra electrons toward uncoordinated Pb ions at the perovskite film surface and grain boundaries, forming strong Pb-F ionic bonds, therefore efficiently passivating the widespread uncoordinated Pb ions defects. Recently, Li et al. employed fluoride to simultaneously passivate both anion and cation vacancies of halide perovskites, by taking the advantage of its high electronegativity [39]. This conclusion agrees well with our previous reports that the incorporation of fluorine-containing functional groups at the perovskite/HTL interface can efficiently passivate the interfacial defects and boost the photovoltaic performances of PSCs [40]. Figure 1(d) presents the results of steady-state photoluminescence (PL) characterization of the deposited CsPbI_2.5_Br_0.5_ thin film and CsPbI_2.5_Br_0.5_/FCQDs GHJ thin films on glass substrates. By formation of graded heterojunction, the PL emission peak changed from ~1.81 eV to ~1.82 eV for the CsPbI_2.5_Br_0.5_ perovskite thin film. A blue-shifted PL emission peak can be correlated with filling of the trap states on CsPbI_2.5_Br_0.5_ layers' surface or along the grain boundaries by injection of FCQDs [40]. Moreover, a strong photoluminescence quenching of ~60% was observed in the CsPbI_2.5_Br_0.5_/FCQDs GHJ thin film as a result of charge transfer from CsPbI_2.5_Br_0.5_ to FCQDs. This was further confirmed by time-resolved PL decay characterization, Figure 1(e). A considerably reduced PL decay lifetime (from ~21 to 11 ns) for the CsPbI_2.5_Br_0.5_/FCQDs GHJ sample demonstrates substantial photogenerated charge carriers transfer from CsPbI_2.5_Br_0.5_ to FCQDs.

**Figure 2:**
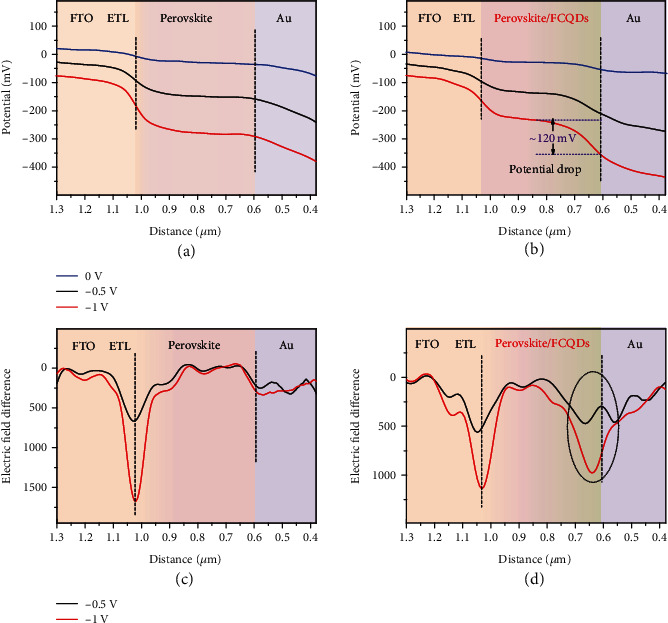
Scanning Kevin probe force microscopy characterization of the graded heterojunction. Cross-sectional potential distribution of the samples (FTO/TiO_2_/CsPbI_2.5_Br_0.5_/Au, sample (a), and FTO/TiO_2_/CsPbI_2.5_Br_0.5_/FCQDs GHJ/Au, sample (b)) under different bias voltages. The calculated electric field distribution of the corresponding samples under different bias voltages of the samples (FTO/TiO_2_/CsPbI_2.5_Br_0.5_/Au, sample (c), and FTO/TiO_2_/CsPbI_2.5_Br_0.5_/FCQDs GHJ/Au, sample (d)).

To gain more insight about the construction of CsPbI_2.5_Br_0.5_/FCQDs GHJ on charge transfer, we investigated the electrical potential distribution across the GHJ structure by performing scanning Kevin probe force microscopy (KPFM) characterization on cross-section of the FTO/TiO_2_/CsPbI_2.5_Br_0.5_/Au and the FTO/TiO_2_/CsPbI_2.5_Br_0.5_/FCQDs GHJ/Au samples in dark condition as shown in Figure S5 [41–44]. To exclude the interference of surface charges on the cleaved surface, the potential distribution measurements were performed at different biases voltage (0 V, −0.5 V, and −1.0 V) [42]. Figures 2(a) and 2(b) present the averaged potential profiles across the two samples. In contrast to the FTO/TiO_2_/CsPbI_2.5_Br_0.5_/Au sample which exhibited a relatively constant potential distribution across the perovskite absorption layer, a potential drop of about 120 mV was observed at the perovskite/FCQDs graded heterojunction area at −1.0 V bias voltage in the FTO/TiO_2_/CsPbI_2.5_Br_0.5_/FCQDs GHJ/Au sample. This obvious potential drop can be caused by charge diffusion motion at the perovskite/FCQDs interface, which also demonstrates the existence of depletion region at the perovskite/FCQDs heterojunction interface. The electric field distribution profiles can be determined as shown in Figures 2(c) and 2(d) by taking the first derivative of the potential differences (Figure S6) for the samples. For the control sample, the electric field at the perovskite/Au interface is quite weak, which implies no obvious depletion region at the interface. Therefore, the photo-generated holes need to diffuse through the whole perovskite absorption layer to be collected by the Au electrode. In contrast, a strong and wide nonzero electric field was identified at the perovskite/FCQDs graded heterojunction area, indicating the formation of a wide depletion region with strong built-in electric field across the junction. This wide depletion region across the perovskite/FCQDs graded heterojunction is expected to facilitate photogenerated hole extraction and thus decrease charge carrier recombination loss.

**Figure 3:**
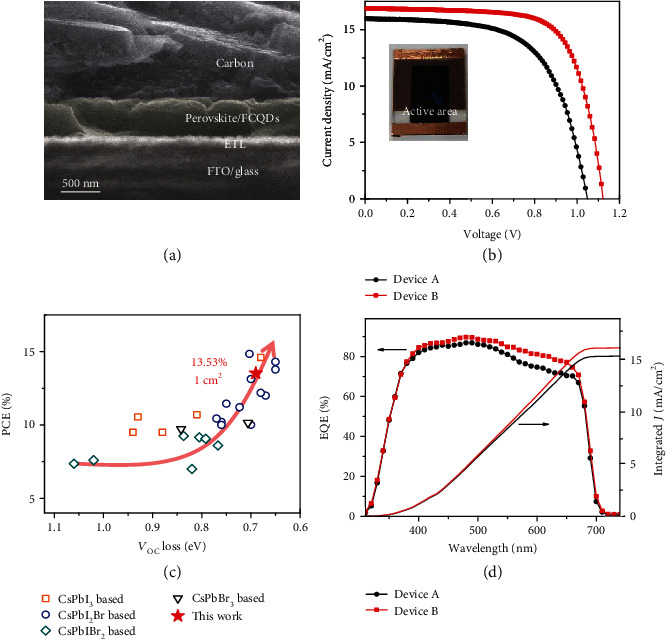
Photovoltaic performance of the graded heterojunction devices. (a) Cross-sectional SEM image of FTO/TiO_2_/CsPbI_2.5_Br_0.5_/FCQDs GHJ/carbon completed perovskite device. (b) Current density-Voltage measurements of the 1.0 cm^2^ perovskite devices. The inset shows an optical photograph of a perovskite device. (c) A brief summary of perovskite devices that using inorganic perovskites as a light absorber and carbon counter-electrode as current collector reported so far. (Values of *V*_OC_ loss and PCE are taken from literature, Table S1, Supporting Information). (d) EQE spectra and integrated current density of the perovskite devices.

We further fabricated CsPbI_2.5_Br_0.5_/FCQDs GHJ PSCs using low-temperature processed carbon electrode as current collector. With the merit of low-temperature large-area processability and low manufacture cost, the carbon electrode based CsPbI_2.5_Br_0.5_/FCQDs GHJ PSCs can be easily scaled up for practical applications.  Figure 3(a) presents the cross-sectional SEM image of a completed perovskite/FCQDs GHJ device, from which a dense and uniform CsPbI_2.5_Br_0.5_/FCQDs film can be clearly observed.  Figure 3(b) presents photocurrent density-voltage (*J* − *V*) measurement curves of the optimized 1.0 cm^2^ PSC devices. We observed that the PSC device (device B) based on perovskite/FCQDs GHJ exhibited improved photovoltaic performance. The champion device showed a *V*_OC_ of 1.12 V, a *J*_SC_ of 16.87 mA cm^−2^, a FF of 71.6%, yielding an impressive PCE of 13.53%. This is one of the highest PCE among carbon electrode-based inorganic PSCs reported so far. In contrast, the control device (device A) showed a total PCE of 10.70%, with a *V*_OC_ of 1.05 V, a *J*_SC_ of 15.97 mA cm^−2^, and a FF of 63.8%.  Figure 3(c) presents the plot of PCE versus *V*_OC_ loss for currently reported PSCs (detail data can be found in Table S1) that using inorganic perovskites (CsPbI_3_, CsPbI_2_Br, CsPbIBr_2_, and CsPbBr_3_) as light absorber and carbon counter electrode as current collector. Apparently, our present fabricated CsPbI_2.5_Br_0.5_/FCQDs GHJ device exhibited both high PCE and low *V*_OC_ loss. This result substantiates the promising potential for constructing perovskite/FCQDs GHJ toward highly efficient PSCs. To the most of our knowledge, this is the first report of 1 cm^2^ carbon counter electrode based inorganic PSC with PCE exceeding 13%.  Figure 3(d) compares the results of external quantum efficiency (EQE) characterization of the aforementioned PSC devices. Device A exhibited obvious EQE drop in long wavelength region (>600 nm), implying inefficient collection of charge carriers generated in the region close to the back contact of the absorber layer [45–47]. For the HTL-free PSCs, severe charge transfer barrier usually exists at the perovskite/counter electrode interface, thus decreasing charge collection efficiency in such region of the devices [45]. Impressively, device B based on perovskite/FCQDs GHJ exhibited much-improved EQE response in the long wavelength region, which confirms the augmented charge collection efficiency. The integrated *J*_SC_ calculated from the EQE spectra for devices A and B were estimated to be 15.40 and 16.19 mA cm^−2^, respectively.

**Figure 4:**
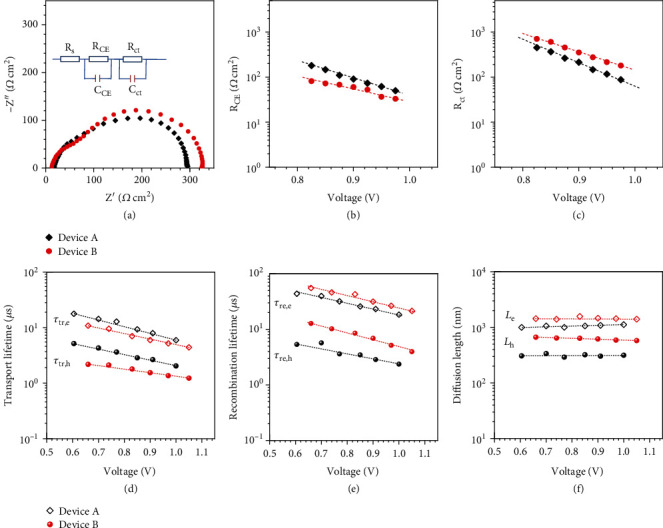
Interfacial charge transfer kinetics of the graded heterojunction devices. (a) Electronic impedance spectroscopy measurements of the devices under 10 mWcm^−2^ illumination at a bias voltage of 0.9 V. (b) Charge transport resistance (*R*_CE_) and (c) charge recombination resistance (*R*_CT_) as a function of the applied bias voltage obtained from impedance measurements. (d) Transport lifetime (*τ*_tr_) and (e) recombination lifetime (*τ*_re_) of the devices obtained from transient photovoltage/photocurrent decay measurements. (f) The calculated diffusion length of devices.

Electronic impedance spectroscopy (EIS) measurements were conducted to understand the effect of perovskite/FCQDs GHJ on electronic processes at the perovskite/carbon electrode interface. The measurements were performed by connecting the FTO glass as working electrode and carbon electrode as counter electrode and reference electrode [48–52].  Figure 4(a) presents the result of EIS characterization in the form of Nyquist plots when the devices were biased at 0.9 V under 10 mW cm^−2^ illumination. Two semicircles were observed in the frequency analysis. The first arc corresponds to charge transport processes in the charge transport layers or at the charge transport layer/counter-electrode interfaces in the high-frequency region, i.e., fast charge transport processes. The second arc can be assigned to charge recombination processes at the perovskite layer/charge selective contact interfaces in the intermediate frequency region, i.e., relative slow charge transport processes [49]. We fitted the EIS plots with equivalent circuit exhibited in the inset of Figure 4(a).  Figure 4(b) shows that device B presents smaller charge-transfer resistance (*R*_CE_) at the perovskite layer/counter-electrode interface, which indicates faster transport of holes from the perovskite absorber to the carbon electrode.  Figure 4(c) presents the fitted interfacial charge recombination resistance (*R*_CT_) as a function of bias voltage. Apparently, device B exhibits larger *R*_CT_ under a series of bias voltage, indicating a slower interfacial charge recombination process. Moreover, a relatively smaller capacitance was calculated for device B from the response in the intermediate frequency region (Figure S7), implying less charge carrier accumulation at the perovskite layer/carbon electrode interface and thus the restrain of interfacial charge recombination [52].

To examine the effect of perovskite/FCQDs GHJ on dynamics of charge carrier transfer and recombination, the PSC devices were further characterized with transient photocurrent/photovoltage decay (TPC/TPV) measurements at various bias light intensities (Figure S8). The working principles for measurements of the TPC/TPV have been described in our previous reports [53–55]. By varying the bias light intensity, the corresponding recombination lifetime (*τ*_re_) at a different open-circuit voltage (or quasi-Fermi level) can be obtained in the model of TPV. Similarly, the apparent transport lifetime (*τ*_tr_) is collected at short circuit conditions in TPC measurements. The transport/recombination kinetics in PSCs are frequently modeled with biphasic decay (Figure S8), corresponding to the dynamics of electron and hole, respectively.  Figure 4(d) exhibits the carrier transport lifetime as a function of *V*_OC_ (determined by the incident light intensity) for the devices by fitting the TPC curves. Notably, both electron and hole transport lifetimes of device B are significantly shorter than that of device A. At an identical voltage of ~0.9 V, the transport lifetime of electron (*τ*_tr,e_) of device B is approximately 6 *μ*s, being shorter than that of device A (~8 *μ*s). Similarly, the transport lifetime of hole (*τ*_tr,h_) of device B is approximately 1.5 *μ*s, being shorter than that of device A (~2.6 *μ*s). This result can be attributed to the CsPbI_2.5_Br_0.5_/FCQDs GHJ, which introduces additional driving force for hole transfer, therefore an enhanced charge carrier transport. This result also indicates an imbalanced charge transport in both devices. A similar behavior has been found in the TPV measurements (Figure 4(e)), showing that device B exhibited a longer carrier recombination lifetime (*τ*_re_) of electron and hole compared to device A. At an identical voltage of ~0.9 V, the recombination lifetime of electron (*τ*_re,e_) of device B is approximately 31.5 *μ*s, being longer than that of device A (~23 *μ*s). Similarly, the recombination lifetime of the hole (*τ*_re,h_) of device B is approximately 7 *μ*s, being longer than that of device A (~2.9 *μ*s). A long interfacial recombination lifetime guarantees efficient charge collection and thus enhancing the device output photovoltage. The diffusion length (*L*) of charge carriers in CsPbI_2.5_Br_0.5_ PSCs was further evaluated by the ratio of recombination lifetime and transport lifetime, as given by the expression *L* = (*D* × *τ*_re_)^1/2^, where *D* is the charge carrier diffusion coefficient obtained from charge transport time *τ*_tr_. As shown in Figure 4(f), device B has a longer charge diffusion length (*L*). At an identical voltage of ~0.9 V, the diffusion length of electron (*L*_*e*_) of device B is approximately 1.46 *μ*m, being longer than that of device A (~1.06 *μ*m). The diffusion length of the hole (*L*_*h*_) of device B is approximately 617 nm, being longer than that of device A (~303 nm). In comparison with previous report on CsPbI_2_Br-based inorganic PSC showing diffusion length of 463 nm for electrons and 202 nm for holes, in this study, the CsPbI_2.5_Br_0.5_/FCQDs GHJ device exhibited much longer diffusion length for both electrons and holes [55]. Especially, such a greatly increased hole diffusion length indicates effective hole collection efficiency, which principally delivers higher output photocurrent. This result also well explains the increased EQE value observed for device B in long wavelength region (Figure 3(d)), which can be ascribed to the better charge carrier collection efficiency. The negative slope of the EQE curve for both devices towards longer wavelengths could be due to the loss of carriers by recombination in the bulk of the perovskite layer. Clearly, we should concern to increase the hole diffusion length along with a reduced interfacial charge recombination with the aim to a balanced charge transport and further improvement in device performace.

**Figure 5:**
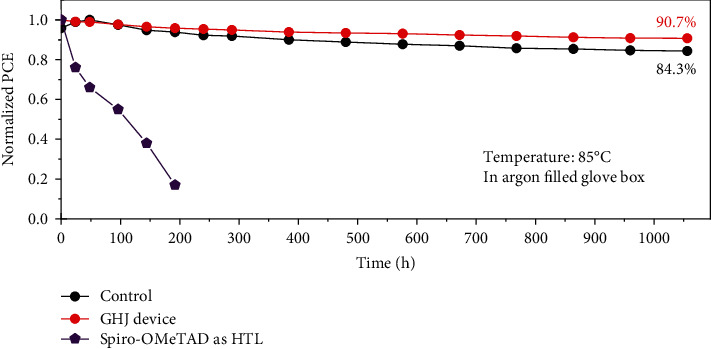
Stability of the graded heterojunction devices. Long-term stability test of unencapsulated PSC devices under 85°С thermal aging in argon-filled atmosphere.

Long-term stability is another momentous metrics for the practical application of PSCs [56]. To estimate long-term stability of the carbon electrode-based CsPbI_2.5_Br_0.5_ perovskite/FCQDs GHJ PSCs, the unencapsulated CsPbI_2.5_Br_0.5_ devices (control) and CsPbI_2.5_Br_0.5_/FCQDs GHJ devices were measured under 85°C thermal-aging condition in argon-filled glove box. Herein, 85°C was selected for thermal-aging according to the standard IEC 61215-1 : 2016 [57], and the inert atmosphere (argon) was chosen to investigate the intrinsic stability of PSCs by eliminating the interference of other stresses from the ambient [58, 59]. The PSC device based on commonly used spiro-OMeTAD as HTL and Au as counter-electrode was measured under identical conditions as a reference. As shown in Figure 5, after thermal aging at 85°С for 1056 hours, the carbon electrode-based CsPbI_2.5_Br_0.5_ devices (control) maintained 84.3% of the initial efficiency, while the CsPbI_2.5_Br_0.5_/FCQDs GHJ device maintained 90.7% of the initial efficiency. In contrast, the PSC device using spiro-OMeTAD as HTL and Au as counter-electrode almost lose 100% of the initial efficiency within 200 hours. The considerable improvement in long-term thermal stability of the carbon electrode-based CsPbI_2.5_Br_0.5_/FCQD GHJ devices can be attributed to the elimination of organic HTL and metal-electrode, as well as the robust shielding effect of FCQDs. These results demonstrate the significance of constructing carbon electrode-based inorganic perovskite/FCQDs GHJ PSCs for achieving long-term stability and provide important direction to realize low cost and highly stable inorganic PSCs toward commercialization.

## 3. Discussion

In summary, we first present a compositionally graded heterojunction structure for photovoltaic application by injecting fluorine-doped carbon quantum dots into inorganic perovskite CsPbI_2.5_Br_0.5_. This CsPbI_2.5_Br_0.5_/FCQDs GHJ can effectively facilitate the separation of photo-generated charge carriers and their transport in photovoltaic devices. Using this novel inorganic perovskite/FCQDs graded heterojunction in conjunction with low-temperature processed carbon electrode, a PCE of 13.53% can be achieved over 1 cm^2^ device. Moreover, the carbon electrode based CsPbI_2.5_Br_0.5_/FCQDs GHJ PSCs exhibited excellent long-term stability, maintaining 90.7% of the initial efficiency under 85°С thermal aging for 1056 hours. This inorganic perovskite/FCQDs graded heterojunction offers a visible pathway to solve charge extraction obstacle in large-area carbon electrode based inorganic PSCs and provides an important direction to realize low-cost and highly stable inorganic PSCs toward commercialization.

## 4. Materials and Methods

Synthesis of fluorine-doped carbon quantum dots (FCQDs): a one-step hydrothermal method was adapted to prepare FCQDs [60]. Briefly, 360 mg of citric acid, 1080 mg of urea, and 200 mg of sodium fluoride were added in 20 mL deionized water. The mixture was sonicated for about 30 min and transferred into a Teflon-lined autoclave. After heating at 180°C for 10 h and naturally cooling, the obtained FCQDs were purified by dialysis against distilled water. Then, the solution was centrifuged at 10000 rpm, and the obtained FCQDs were dispersed in chlorobenzene for use.

PSC devices fabrication: the compact TiO_2_ layer was prepared by spray pyrolysis deposition of titanium isopropoxide ethyl alcohol solution, followed by annealing at 100°C for 10 min and 450°C for 30 min. The mesoporous TiO_2_ layer was prepared by spin-coating of 18 nm particle size paste (Dyesol 18 NR-D) diluted in ethanol at 3000 for rpm 30 s, then sintered at 100°C for 10 min and 450°C for 1 hour. The 0.8 M CsPbI_2.5_Br_0.5_ perovskite precursor solution was made by dissolving CsI, CsBr, and PbI_2_ (molar ratio 1 : 1 : 2) in a mixture of DMF and DMSO (*v*/*v*, 4 : 1) and stirred overnight. For the graded heterojunction device, 2 mg/mL FCQDs were added into chlorobenzene antisolvent and stirred overnight. The perovskite solution was spin-coated onto FTO/TiO_2_ substrates at 2000 rpm for 10 s and 6000 rpm 30 s, and chlorobenzene with or without FCQDs was dropped onto the film at the last 23 s during the second step. Subsequently, the spin-coated perovskite films were dried for 5 min, then thermally annealed at 70°C for 3 min and 300°C for 10 min. Finally, the carbon paste was coated by the doctor-blade method and annealed at 100°C for 30 min [61].

Other experimental details and characterizations are shown in the Supporting Information.

## Data Availability

The experimental data is available if required.

## Supplementary Materials

Supplementary Materials Materials and experimental details are given in supporting information. Figure S1: (a) the chemical structure of fluorine-doped carbon quantum dots (FCQDs). (b) TEM and HRTEM (inset) images of FCQDs. (c) Diameter distribution of the presynthesized FCQDs from the TEM image measured using the Nano Measurer software. (d) XPS survey spectra of FCQDs. (e) Detailed C 1 s XPS spectra of FCQDs. (f) Detailed F 1 s XPS spectra of FCQDs. Figure S2: the derived curves from UV-vis absorption spectra for (a) FCQDs and (b) CsPbI_2.5_Br_0.5_ perovskite films for bandgap evaluation. (c) The curves of ultraviolet photoelectron spectroscopy (UPS) measurement on CsPbI_2.5_Br_0.5_ perovskite and FCQDs samples. (d) The calculated *E*_VB_, *E*_CB_ and *E*_*F*_ of CsPbI_2.5_Br_0.5_ perovskite and FCQDs. Figure S3: SEM images of control perovskite thin film and perovskite/FCQDs GHJ thin film. Figure S4: Pb 4f XPS spectra of control perovskite thin film and perovskite/FCQDs GHJ thin film. Figure S5: schematic diagram of scanning Kevin probe force microscopy characterization. Figure S6: calculated potential differences of the samples with respect to the 0 V curve. Figure S7: corresponding capacitance (C) as a function of the applied voltage at open-circuit condition obtained from impedance measurements. Figure S8: transient photocurrent and transient photovoltage decay curves of the devices under varying light intensity. The solid lines are fitted curves using a biexponential equation. Figure S9: current density-voltage measurement curves and PCE distribution of carbon electrode-based CsPbI_2.5_Br_0.5_/FCQDs planar heterojunction PSCs. Table S1: a brief summary of perovskite solar cells that using inorganic perovskites as light absorber and carbon counter electrode as current collector.
