# Supplementary material for: Efficient and Stable Large-Area Perovskite Solar Cells with Inorganic Perovskite/Carbon Quantum Dot-Graded Heterojunction
Source: Research (Wash D C). 2021 Jul 12;2021:9845067. doi: 10.34133/2021/9845067 (PMC8292841; doi:10.34133/2021/9845067)
Supplement: Supplementary Materials — Materials and experimental details are given in supporting information. Figure S1: (a) the chemical structure of fluorine-doped carbon quantum dots (FCQDs). (b) TEM and HRTEM (inset) images of FCQDs. (c) Diameter distribution of the presynthesized FCQDs from the TEM image measured using the Nano Measurer software. (d) XPS survey spectra of FCQDs. (e) Detailed C 1 s XPS spectra of FCQDs. (f) Detailed F 1 s XPS spectra of FCQDs. Figure S2: the derived curves from UV-vis absorption spectra for (a) FCQDs and (b) CsPbI2.5Br0.5 perovskite films for bandgap evaluation. (c) The curves of ultraviolet photoelectron spectroscopy (UPS) measurement on CsPbI2.5Br0.5 perovskite and FCQDs samples. (d) The calculated EVB, ECB and EF of CsPbI2.5Br0.5 perovskite and FCQDs. Figure S3: SEM images of control perovskite thin film and perovskite/FCQDs GHJ thin film. Figure S4: Pb 4f XPS spectra of control perovskite thin film and perovskite/FCQDs GHJ thin film. Figure S5: schematic diagram of scanning Kevin probe force microscopy characterization. Figure S6: calculated potential differences of the samples with respect to the 0 V curve. Figure S7: corresponding capacitance (C) as a function of the applied voltage at open-circuit condition obtained from impedance measurements. Figure S8: transient photocurrent and transient photovoltage decay curves of the devices under varying light intensity. The solid lines are fitted curves using a biexponential equation. Figure S9: current density-voltage measurement curves and PCE distribution of carbon electrode-based CsPbI2.5Br0.5/FCQDs planar heterojunction PSCs. Table S1: a brief summary of perovskite solar cells that using inorganic perovskites as light absorber and carbon counter electrode as current collector. [file 9845067.f1.pdf]

Supporting Information

Efficient and Stable Large-area Perovskite Solar Cells with Inorganic Perovskite/carbon quantum Dots Graded Heterojunction

Qiang Sun[1], Cai Shen[2], Deyu Wang[2], Tao Zhang[1], Huaxia Ban[1], Yan Shen[1], Zhipan Zhang[3], Xiao-Li Zhang[4], Guanjun Yang[5], Mingkui Wang [1]*

[1] Wuhan National Laboratory for Optoelectronics, Huazhong University of Science and Technology, Wuhan, Hubei 430074, P. R. China.

[2] Ningbo Institute of Materials Technology & Engineering, Chinese Academy of Sciences, 1219 Zhongguan Road, Ningbo 315201, P. R. China.

[3] School of Chemistry, Beijing Institute of Technology, Beijing, 102488, P. R. China.

[4] State Centre for International Cooperation on Designer Low-Carbon & Environmental Materials, School of Materials Science and Engineering, Zhengzhou University, 450001, P. R. China.

[5] State Key Laboratory for Mechanical Behavior of Materials, Xi'an Jiaotong University, Xi'an, 710049, P. R. China.

E-mail: mingkui.wang@mail.hust.edu.cn

Materials

CsI (99.9%, Sigma Aldrich), CsBr (99.99%, Sigma Aldrich), PbI_2 (99.999%, Alfa Aesar), N, N-Dimethylformamide (DMF, Anhydrous 99.8%, Sigma Aldrich), dimethyl sulfoxide (DMSO, Anhydrous ≥ 99.5%, Sigma Aldrich), Chlorobenzene (CB, Anhydrous 99.8%, Sigma Aldrich), Citric acid (99.5%, aladdin), urea (99.999%, aladdin), and sodium fluoride (99.99%, aladdin) were used as received.

Characterization

X-ray diffraction (XRD) experiments were conducted using a Philips X-ray diffractometer with Cu Kα radiation. UV-visible absorption spectra were observed with PerkinElmer LAMBDA 950 spectrophotometer. XPS and UPS measurements were performed on a Kratos AXIS Ultra-DLD ultra-high-vacuum photoemission spectroscopy system. Top view and cross section SEM images of the samples were obtained with a field-emission scanning electron microscope, FEI Nova NanoSEM 450. HRTEM analyses were carried out on FEI Tecnai G^2 20 microscope. Time-of-flight secondary ion mass spectrometry (ToF-SIMS, TOF.SIMS 5-100, Germany) was used to detect the depth profiles of the involved species. Steady-state photoluminescence (PL) spectra were performed using the fluorescence spectrophotometer (LabRAM HR800). Time-resolved photoluminescence decay measurements were performed at 680 nm with a 478 nm light pulse as excitation by Delta Flex Fluorescence Lifetime System (Horiba Scientific Com., Japan). A xenon light source solar simulator (450 W, Oriel, model 9119) with an AM 1.5 G filter (Oriel, model 91192) was used to give an irradiance of $100 mWcm^{-2}$ at the surface of the solar cells. The photocurrent density-voltage (J-V) characteristics of the perovskite solar cell devices were measured by recording the current through Keithley 2400 digital source meter. A similar data acquisition

system was used to control the incident photon-tocurrent conversion efficiency (EQE) measurement. The electronic impedance measurements were performed using the PGSTAT302N frequency analyzer from Autolab (The Netherlands) together with the Frequency Response Analyzer to give voltage modulation under the giving range of frequency.

Surface potential was measured by Kevin probe force microscopy (KPFM, Bruker dimension icon) in argon filled glove box at room temperature. The samples were glue to the sample stage by conductive silver paste. The morphology and contact potential difference (CPD) of the sample (cross sectional, from gold layer, perovskite layer, ETL layer to FTO layer) was collected in AM-KPFM mode by a conductive Pt/Ir coated probes (tip model: SCM-PIT with k=0.28 N/m, Bruker Corporation). Image scan rate was set to be 1 Hz per line with a resolution of 256×256 pixels. The lift height was set to be 100 nm. Surface potential distributions were acquired under sample bias of 0 V, −0.5 V, and −1 V respectively.

The transient photovoltage/photocurrent decay measurements were performed on sample devices using 532 nm Green Solid-State Lasers (AO-S-532-100uJ) to generate a perturbation pulse with a width of 20 ns. An array of InGaN diodes (Lumiled) supplied the white bias light illumination. Transient decays were measured at different white light intensities via tuning the voltage applied to the bias diodes. The pulsed light and steady-state white-lights were both incident on the transparent side of a testing cell. The pulsed lights were carefully controlled by the driving potential of the laser to keep the modulated photovoltages below 26 mV (HuaMing, model 201703).

Table S1. A brief summary of perovskite solar cells that using inorganic perovskites as light absorber and carbon counter electrode as current collector.

Cell Structure	V_{OC} (V)	V_{OC} loss (V)	J_{SC} (mA/cm^2)	FF (%)	PCE (%)	Ref.
FTO/TiO_2/$CsPbI_2Br$ /P3HT/MWCNT/Carbon	1.21	0.70	13.35	62.00	10.01	[1]
FTO/c-TiO_2/$CsPbI_2Br$/Carbon	1.15	0.76	13.87	64.00	10.21	[2]
FTO/ZnO/$CsPbIBr_2$/Carbon	1.03	1.02	11.60	63.00	7.60	[3]
FTO/c-TiO_2/$CsPbIBr_2$/Carbon	1.245	0.805	10.66	69.00	9.16	[4]
FTO/c-TiO_2/$CsPbI_2Br$/Carbon	1.15	0.76	13.54	64.20	10.00	[5]
ITO/SnO_2/$CsPbI_2Br$/Co_3O_4/Carbon	1.187	0.723	13.09	72.12	11.21	[6]
FTO/Nb_2O_5/$CsPbI_2Br$/Carbon	1.24	0.67	14.02	69.00	12.00	[7]
FTO/c-TiO_2 /$CsPbIBr_2$/Carbon	1.283	0.767	11.17	60.00	8.60	[8]
FTO/c-TiO_2/CsBr/$CsPbIBr_2$/Carbon	1.258	0.792	11.61	62.00	9.06	[9]
FTO/SnO_2/$CsPbI_2Br$ (with PbSCN)/Carbon	1.14	0.77	14.25	64.12	10.44	[10]
ITO/SnO_2/$CsPbIBr_2$/Carbon	1.23	0.82	8.50	67.00	7.00	[11]
FTO/c-TiO_2/$CsPbIBr_2$ (Li doping)/CuPc/Carbon	1.213	0.837	10.27	74.30	9.25	[12]
FTO/c-TiO_2/m-TiO_2/$CsPbIBr_2$ (Mn doping)/Carbon	0.99	1.06	13.15	57.00	7.36	[13]
FTO/c-TiO_2/m-TiO_2/$CsPbBr_3$/Carbon	1.458	0.842	8.12	82.10	9.72	[14]
FTO/c-TiO_2/m-TiO_2/$CsPbBr_3$ (Sm doping)/Carbon	1.594	0.706	7.48	85.10	10.14	[15]
FTO/c-TiO_2/m-TiO_2/$CsPbI_3$/Carbon	0.79	0.94	18.50	65.00	9.50	[16]
FTO/c-TiO_2/Cs-TiO_2 nanorods/$CsPbI_3$/Carbon	0.85	0.88	17.80	63.00	9.50	[17]
FTO/c-TiO_2/m-TiO_2/$CsPbI_3$ (Na doping)/Carbon	0.92	0.81	16.50	70.30	10.70	[18]
ITO/SnO_2/$CsPbI_2Br$/ Carbon	1.23	0.68	15.46	64.00	12.19	[19]
ITO/c-TiO_2/m-TiO_2/$CsPbI_2Br$/ Carbon	1.21	0.70	14.94	72.59	13.13	[20]
FTO/c-TiO_2/m-TiO_2/$CsPbI_3$/Carbon	1.05	0.68	17.47	79.00	14.60	[21]
FTO/ TiO_2 nanorods/$CsPbI_2Br$/Carbon	1.15	0.75	14.39	69.10	11.45	[22]
ITO/SnO_2/$CsPbI_2Br$/ Carbon	1.26	0.65	14.74	0.74	13.78	[23]
FTO/c-TiO_2/$CsPbI_2Br$/ Carbon	1.26	0.65	14.1	0.806	14.3	[24]
FTO/c-TiO_2/$CsPbI_2Br$/ Carbon	1.207	0.703	16.62	0.74	14.84	[25]
FTO/c-TiO_2/m-TiO_2/ ($CsPbI_{2.5}Br_{0.5}$/FCQDs GHJ)/Carbon	1.12	0.69	16.87	71.60	13.53	This work

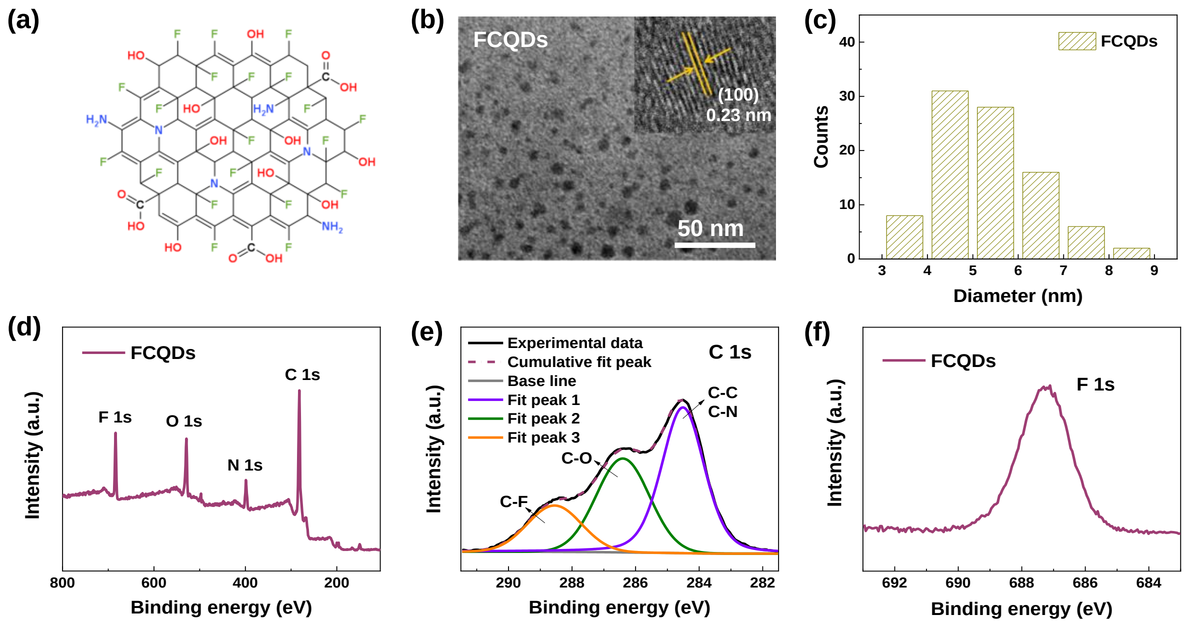

Figure S1. (a) The chemical structure of fluorine doped carbon quantum dots (FCQDs). (b) TEM and HRTEM (inset) images of FCQDs. (c) Diameter distribution of the pre-synthesized FCQDs from the TEM image measured using Nano Measurer. (d) XPS survey spectra of FCQDs. (e) Detailed C 1s XPS spectra of FCQDs. (f) Detailed F 1s XPS spectra of FCQDs.

Transmission electron microscopy (TEM) characterization (Figure S1b) clearly shows that the as-prepared FCQDs are evenly distributed with diameters of 3-7 nm (evaluated with Nano Measurer). The high-resolution TEM image in the inset of Figure S1b reveals a d-spacing value of 0.23 nm, corresponding to the (100) facet of graphitic carbon. [26] X-ray photoelectron spectroscopy (XPS) characterization was conducted to explore the element composition of FCQDs. As shown in Figure S1d, the as-prepared FCQDs are mainly composed of C, N, O, and F, the binding energy at 284.5, 397.4, 531.2, and 687.3 eV are attributed to C 1s, N 1s, O 1s, and F 1s, respectively.

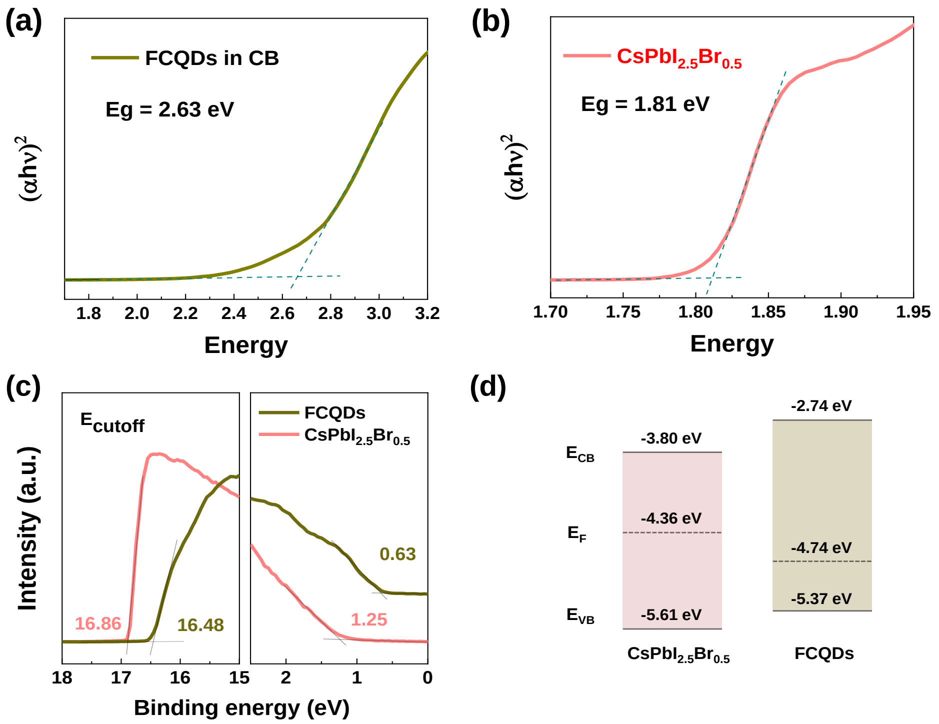

Figure S2. The derived curves from UV-vis absorption spectra for (a) FCQDs and (b) $CsPbI_{2.5}Br_{0.5}$ perovskite films for bandgap evaluation. (c) The curves of ultraviolet photoelectron spectroscopy (UPS) measurement on $CsPbI_{2.5}Br_{0.5}$ perovskite and FCQDs samples. (d) The calculated E_{VB}, E_{CB} and E_F of $CsPbI_{2.5}Br_{0.5}$ perovskite and FCQDs.

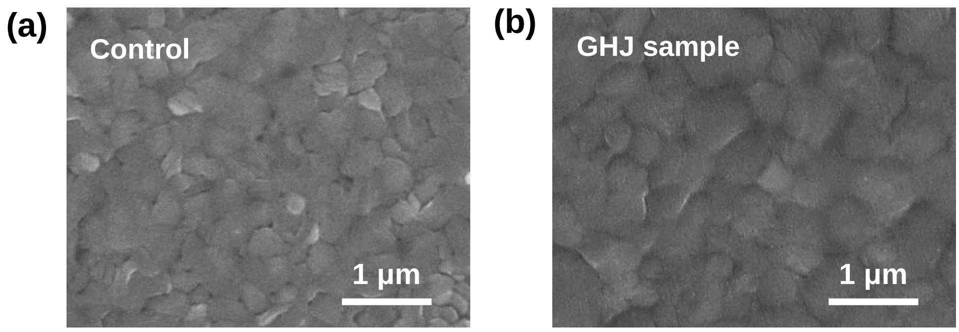

Figure S3. SEM images of control perovskite thin film and perovskite/FCQDs GHJ thin film.

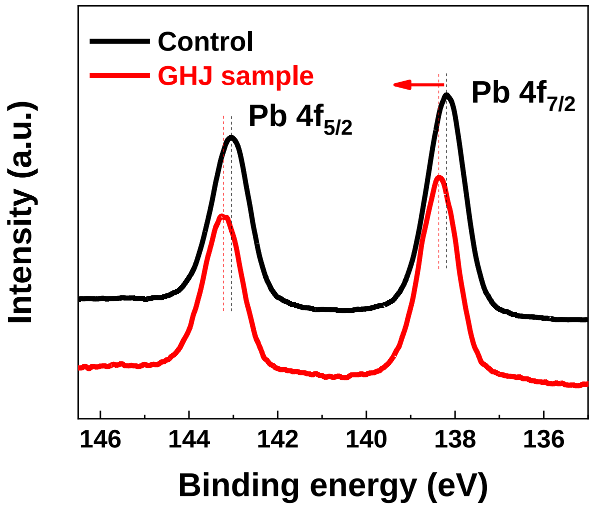

Figure S4. Pb 4f XPS spectra of the samples (FTO/TiO_2/$CsPbI_{2.5}Br_{0.5}$/Au and FTO/TiO_2/$CsPbI_{2.5}Br_{0.5}$/FCQDs GHJ/Au).

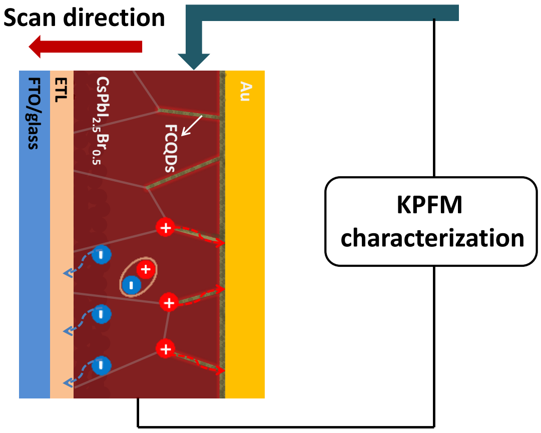

Figure S5. Schematic diagram of scanning Kevin probe force microscopy characterization.

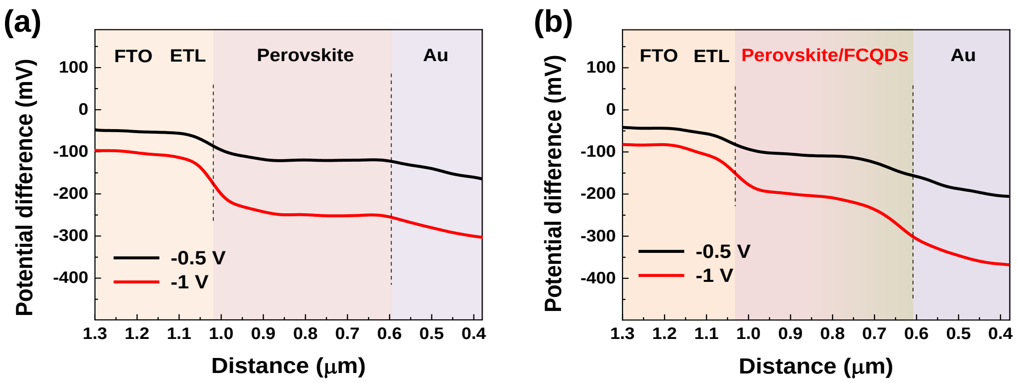

Figure S6. Calculated potential differences of the samples with respect to the 0 V curve.

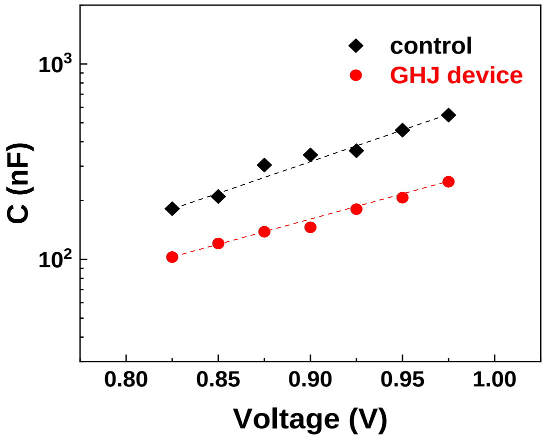

Figure S7. Corresponding capacitance (C) as a function of the applied voltage at open-circuit condition obtained from impedance measurements.

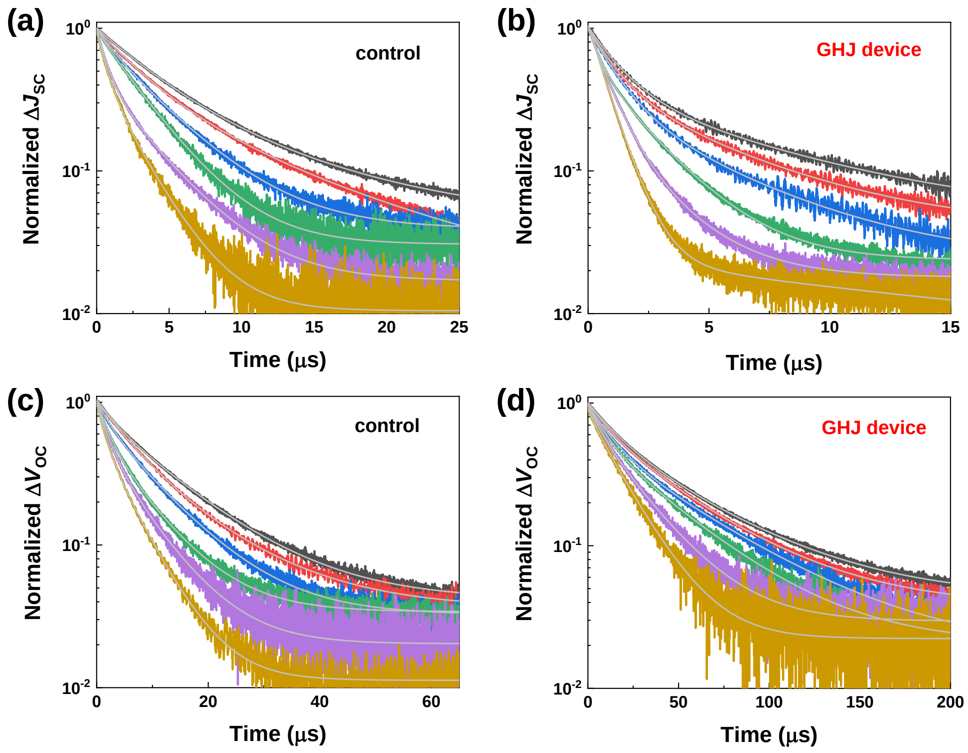

Figure S8. Transient photocurrent and Transient photovoltage decay curves of the devices under varying light intensity. The solid lines are fitted curves using a bi-exponential equation.

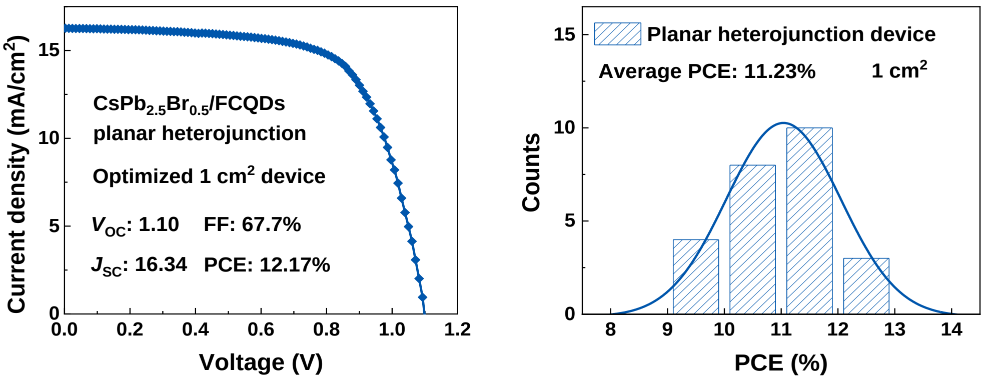

Figure S9. Current density-voltage measurement curves and PCE distribution of carbon-electrode based $CsPbI_{2.5}Br_{0.5}$/FCQDs planar heterojunction PSCs.

We performed additional experiments of carbon-electrode based $CsPbI_{2.5}Br_{0.5}$/FCQDs planar heterojunction PSCs (in which FCQDs was fabricated on the top-layer of $CsPbI_{2.5}Br_{0.5}$ perovskite). The 1 cm^2 PSC devices showed an average PCE of 11.23%, and the optimized device showed a PCE of 12.17%, much better than that of the control device, but inferior to that of the $CsPbI_{2.5}Br_{0.5}$/FCQDs graded heterojunction device. This result demonstrates that FCQDs can promotes photo-generated charge extraction in carbon-electrode based inorganic PSCs, resulting in improved photovoltaic performance.

Reference:

[1] G. Wang, J. Liu, K. Chen, R. Pathak, A. Gurung, Q. Qiao, High-performance carbon electrode-based $CsPbI_2Br$ inorganic perovskite solar cell based on poly(3-hexylthiophene)-carbon nanotubes composite hole-transporting layer, J. Colloid Interface Sci. 555 (2019) 180-186.

[2] C. Dong, X. Han, W. Li, Q. Qiu, J. Wang, Anti-solvent assisted multi-step deposition for efficient and stable carbon-based $CsPbI_2Br$ all-inorganic perovskite solar cell, Nano Energy 59 (2019) 553-559.

[3] C. Wang, J. Zhang, L. Jiang, L. Gong, H. Xie, Y. Gao, H. He, Z. Fang, J. Fan, Z. Chao, All-inorganic, hole-transporting-layer-free, carbon-based $CsPbIBr_2$ planar solar cells with ZnO as electron-transporting materials, J. Alloys Compd. 817 (2020) 152768.

[4] W. Zhu, Q. Zhang, D. Chen, Z. Zhang, Z. Lin, J. Chang, J. Zhang, C. Zhang, Y. Hao, Intermolecular Exchange Boosts Efficiency of Air-Stable, Carbon-Based All-Inorganic Planar $CsPbIBr_2$ Perovskite Solar Cells to Over 9%, Adv. Energy Mater. 8 (2018) 1802080.

[5] C. Dong, X. Han, Y. Zhao, J. Li, L. Chang, W. Zhao, A Green Anti-Solvent Process for High Performance Carbon-Based $CsPbI_2Br$ All-Inorganic Perovskite Solar Cell, Sol. RRL 2 (2018) 1800139.

[6] Y. Zhou, X. Zhang, X. Lu, X. Gao, J. Gao, L. Shui, S. Wu, J. Liu, Promoting the Hole Extraction with Co_3O_4 Nanomaterials for Efficient Carbon-Based $CsPbI_2Br$ Perovskite Solar Cells, Sol. RRL 3 (2019) 1800315.

[7] Y. Guo, F. Zhao, J. Tao, J. Jiang, J. Zhang, J. Yang, Z. Hu, J. Chu, Efficient and Hole-Transporting-Layer-Free $CsPbI_2Br$ Planar Heterojunction Perovskite Solar Cells through Rubidium Passivation, ChemSusChem 12 (2019) 983-989.

[8] Q. Zhang, W. Zhu, D. Chen, Z. Zhang, Z. Lin, J. Chang, J. Zhang, C. Zhang, Y. Hao, Light Processing Enables Efficient Carbon-Based, All-Inorganic Planar $CsPbIBr_2$ Solar Cells with High Photovoltages, ACS Appl. Mater. Interfaces 11 (2019) 2997-3005.

[9] W. Zhu, Z. Zhang, W. Chai, D. Chen, H. Xi, J. Chang, J. Zhang, C. Zhang, Y. Hao, Benign Pinholes in $CsPbIBr_2$ Absorber Film Enable Efficient Carbon-Based, All-Inorganic Perovskite Solar Cells, ACS Appl. Energy Mater. 2 (2019) 5254-5262.

[10] Z. Ye, J. Zhou, J. Hou, F. Deng, Y. Zheng, X. Tao, Low Temperature-Processed Stable and Efficient Carbon-Based $CsPbI_2Br$ Planar Perovskite Solar Cells by In Situ Passivating Grain Boundary and Trap Density, Sol. RRL 3 (2019) 1900109.

[11] Z. Guo, S. Teo, Z. Xu, C. Zhang, Y. Kamata, S. Hayase, T. Ma, Achievable high V_{OC} of carbon based all-inorganic $CsPbIBr_2$ perovskite solar cells through interface engineering, J. Mater. Chem. A 7 (2019) 1227-1232.

[12] X. Tan, X. Liu, Z. Liu, B. Sun, J. Li, S. Xi, T. Shi, Z. Tang, G. Liao, Enhancing the optical, morphological and electronic properties of the solution-processed $CsPbIBr_2$ films by Li doping for efficient carbon-based perovskite solar cells, Appl. Surf. Sci. 499 (2020) 143990.

[13] J. Liang, Z. Liu, L. Qiu, Z. Hawash, L. Meng, Z. Wu, Y. Jiang, L. Ono, Y. Qi, Enhancing Optical, Electronic, Crystalline, and Morphological Properties of Cesium Lead Halide by Mn Substitution for High-Stability All-Inorganic Perovskite Solar Cells with Carbon Electrodes, Adv. Energy Mater. 8 (2018) 1800504.

[14] J. Duan, Y. Zhao, B. He, Q. Tang, High-Purity Inorganic Perovskite Films for Solar Cells with 9.72 % Efficiency, Angew. Chem. Int. Ed. 57 (2018) 3787-3791.

[15] J. Duan, Y. Zhao, X. Yang, Y. Wang, B. He, Q. Tang, Lanthanide Ions Doped $CsPbBr_3$ Halides for HTM-Free 10.14%-Efficiency Inorganic Perovskite Solar Cell with an Ultrahigh Open-Circuit Voltage of 1.594 V, Adv. Energy Mater. 8 (2018) 1802346.

[16] S. Xiang, Z. Fu, W. Li, Y. Wei, J. Liu, H. Liu, L. Zhu, R. Zhang, H. Chen, Highly Air-Stable Carbon-Based α-$CsPbI_3$ Perovskite Solar Cells with a Broadened Optical Spectrum, ACS Energy Lett. 3 (2018) 1824-1831.

[17] J. Liu, L. Zhu, S. Xiang, H. Wang, H. Liu, W. Li, H. Chen, Cs-Doped TiO_2 Nanorod Array Enhances Electron Injection and Transport in Carbon-Based $CsPbI_3$ Perovskite Solar Cells, ACS Sustainable Chem. Eng. 7 (2019) 16927-16932.

[18] S. Xiang, W. Li, Y. Wei, J. Liu, H. Liu, L. Zhu, S. Yang, H. Chen, Natrium Doping Pushes the Efficiency of Carbon-Based $CsPbI_3$ Perovskite Solar Cells to 10.7%, iScience 15 (2019) 156-164.

[19] C. Liu, M. Wu, Y. Wu, D. Wang, T. Zhang, Efficient all-inorganic $CsPbI_2Br$ perovskite solar cell with carbon electrode by revealing crystallization kinetics and improving crystal quality, J. Power Sources 447 (2020) 227389.

[20] S. Gong, H. Li, Z. Chen, C. Shou, M. Huang, S. Yang, $CsPbI_2Br$ Perovskite Solar Cells Based on Carbon Black-Containing Counter Electrodes, ACS Appl. Mater. Interfaces 12 (2020) 34882-34889.

[21] H. Wang, H. Liu, Z. Dong, W. Li, L. Zhu, H. Chen. Composition manipulation boosts the efficiency of carbon-based $CsPbI_3$ perovskite solar cells to beyond 14%. Nano Energy 84 (2021) 105881.

[22] W. Cai, Y. Lv, K. Chen, Z. Zhang, Y. Jin, and X. Zhou. Carbon-Based All-Inorganic $CsPbI_2Br$ Perovskite Solar Cells Using TiO_2 Nanorod Arrays: Interface Modification and the Enhanced Photovoltaic Performance. Energy Fuels 34 (2020) 11670-11678.

[23] Q. Han, S. Yang, L. Wang, F. Yu, C. Zhang, M. Wu, T. Ma. The sulfur-rich small molecule boosts the efficiency of carbon-based $CsPbI_2Br$ perovskite solar cells to approaching 14%. Solar Energy 216 (2021) 351-357.

[24] G. Zhang, P. Xie, Z. Huang, Z. Yang, Z. Pan, Y. Fang, H. Rao, X. Zhong. Modification of Energy Level Alignment for Boosting Carbon-Based $CsPbI_2Br$ Solar Cells with 14% Certified Efficiency. Adv. Funct. Mater. (2021) 2011187.

[25] W. Zhu, W. Chai, D. Chen, J. Ma, D. Chen, H. Xi, J. Zhang, C. Zhang, Y. Hao. High-Efficiency (>14%) and Air-Stable Carbon-Based, All-Inorganic $CsPbI_2Br$ Perovskite Solar Cells through a Top-Seeded Growth Strategy. ACS Energy Lett. 6 (2021) 1500-1510.

[26] G. Zuo, A. Xie, J. Li, T. Su, X. Pan, W. Dong, Large Emission Red-Shift of Carbon Dots by Fluorine Doping and Their Applications for Red Cell Imaging and Sensitive Intracellular Ag^+ Detection, J. Phys. Chem. C 121 (2017) 26558-26565.
